# Supplementary material for: Increased Interleukin-11 and Stress-Related Gene Expression in Human Endothelial and Bronchial Epithelial Cells Exposed to Silver Nanoparticles
Source: Biomolecules. 2021 Feb 7;11(2):234. doi: 10.3390/biom11020234 (PMC7915395; doi:10.3390/biom11020234)
Supplement: Supplementary file 1 [file biomolecules-11-00234-s001.pdf]

Supplementary data:

Increased interleukin-11 and stress-related gene expression in human endothelial and bronchial epithelial cells exposed to silver nanoparticles

Jiyoung Jang[1,2,3], Sun Park[4,5,*] and In-Hong Choi[1,2,]**

[1] Department of Microbiology, Yonsei University College of Medicine, Seoul 03722, Republic of Korea
[2] Institute for Immunology and Immunological Diseases, Yonsei University College of Medicine, Seoul 03722, Republic of Korea
[3] Humidifier Disinfectant Health Center, National Institute of Environmental Research, Incheon 22689, Republic of Korea; Jang191111@korea.kr
[4] Department of Biomedical Sciences, The Graduate School, Ajou University, Suwon 16499, Republic of Korea
[5] Department of Microbiology, Ajou University School of Medicine, Suwon 16499, Republic of Korea
* Correspondence: sinsun@ajou.ac.kr; Tel.: +82-31-219-5071
** Correspondence: inhong@yuhs.ac; Tel.: +82-2-2228-1821

Supplememtal Table S1. Classification of increased genes associated with cell death categories

Ontology term	Count
regulation of programmed cell death	37
regulation of cell death	37
negative regulation of programmed cell death	26
negative regulation of cell death	26
death	22
cell death	21
positive regulation of programmed cell death	12
positive regulation of cell death	12
programmed cell death	17
induction of programmed cell death	10

EA.hy926. Significant transcripts were selected when expression values changed by 1.5-fold or greater and while using a t-test with p-value < 0.05.

Supplememtal Table S2. Classification of increased genes associated with cell survival categories

Ontology term	Count
cell proliferation	10
positive regulation of cell proliferation	14
regulation of cell proliferation	20
regulation of smooth muscle cell proliferation	4
positive regulation of smooth muscle cell proliferation	3
positive regulation of endothelial cell proliferation	2
positive regulation of fibroblast proliferation	1
regulation of endothelial cell proliferation	2
regulation of fibroblast proliferation	1
negative regulation of cell proliferation	6
positive regulation vascular endothelial growth factor production	2
regulation of vascular endothelial growth factor production	2

EA.hy926. Significant transcripts were selected when expression values changed by 1.5-fold or greater and while using a t-test with p-value < 0.05.

Supplememtal Table S3. Classification of increased genes associated with inflammation categories

Ontology term	Count
acute inflammatory response	4
inflammatory response	8
activation of plasma proteins involved in acute inflammatory response	2
Cytokines and Inflammatory Response	2

EA.hy926. Significant transcripts were selected when expression values changed by 1.5-fold or greater and while using a t-test with p-value < 0.05.

Supplememtal Table S4. Classification of increased genes associated with apoptosis categories

Ontology term	Count
apoptotic nuclear changes	1
apoptotic mitochondrial changes	2
regulation of apoptosis	37
anti-apoptosis	23
negative regulation of apoptosis	26
positive regulation of apoptosis	12
apoptosis	17
induction of apoptosis	10
DNA damage response, signal transduction resulting in induction of apoptosis	2
induction of apoptosis by intracellular signals	2
Neuropeptides VIP and PACAP inhibit the apoptosis of activated T cells	2
negative regulation of neuron apoptosis	2
regulation of neuron apoptosis	2
induction of apoptosis by extracellular signals	2

EA.hy926. Significant transcripts were selected when expression values changed by 1.5-fold or greater and while using a t-test with p-value < 0.05.

Supplememtal Table S5. Classification of increased genes associated with ROS categories

Ontology term	Count
response to reactive oxygen species	3
oxygen and reactive oxygen species metabolic process	2
response to oxidative stress	8
Oxidative Stress Induced Gene Expression Via Nrf2	4
cadmium ion binding	4
transition metal ion binding	54
zinc ion binding	45
copper ion binding	4
iron ion binding	7
magnesium ion binding	8
cation binding	67
ion binding	67
metal ion binding	65
manganese ion binding	3
calcium ion binding	5

EA.hy926. Significant transcripts were selected when expression values changed by 1.5-fold or greater and while using a t-test with p-value < 0.05.

Supplememtal Table S6. Classification of increased genes associated with cell death categories

Ontology term	Count
regulation of cell death	49
regulation of programmed cell death	49
negative regulation of programmed cell death	36
negative regulation of cell death	36
death	28
cell death	27
programmed cell death	26
positive regulation of cell death	17
positive regulation of programmed cell death	17
induction of programmed cell death	11

BEAS-2B. Significant transcripts were selected when expression values changed by 2-fold.

Supplememtal Table S7. Classification of increased genes associated with cell survival categories

Ontology term	Count
regulation of survival gene product expression	2
B Cell Survival Pathway	2
regulation of cell proliferation	31
positive regulation of cell proliferation	20
cell proliferation	16
negative regulation of cell proliferation	12
regulation of smooth muscle cell proliferation	7
positive regulation of smooth muscle cell proliferation	5
regulation of epithelial cell proliferation	3
positive regulation of epithelial cell proliferation	2
Cadmium induces DNA synthesis and proliferation in macrophages	2
Inhibition of Cellular Proliferation by Gleevec	2

BEAS-2B. Significant transcripts were selected when expression values changed by 2-fold.

Supplememtal Table S8. Classification of increased genes associated with inflammation categories

Ontology term	Count
inflammatory response	16
acute inflammatory response	3
Cytokines and Inflammatory Response	3
Cells and Molecules involved in local acute inflammatory response	3

BEAS-2B. Significant transcripts were selected when expression values changed by 2-fold.

Supplememtal Table S9. Classification of increased genes associated with apoptosis categories

Ontology term	Count
regulation of apoptosis	49
negative regulation of apoptosis	36
anti-apoptosis	29
apoptosis	25
positive regulation of apoptosis	17
induction of apoptosis	11
regulation of neuron apoptosis	5
regulation of anti-apoptosis	5
positive regulation of anti-apoptosis	4
induction of apoptosis by extracellular signals	3
positive regulation of neuron apoptosis	2
PTEN dependent cell cycle arrest and apoptosis	1
TSP-1 Induced Apoptosis in Microvascular Endothelial Cell	2
Neuropeptides VIP and PACAP inhibit the apoptosis of activated T cells	2
apoptotic mitochondrial changes	3

BEAS-2B. Significant transcripts were selected when expression values changed by 2-fold.

Supplememtal Table S10. Classification of increased genes associated with ROS categories

Ontology term	Count
response to reactive oxygen species	8
response to oxidative stress	11
Oxidative Stress Induced Gene Expression Via Nrf2	3
cellular response to oxidative stress	2
ion binding	74
magnesium ion binding	10
metal ion binding	74
transition metal ion binding	58
zinc ion binding	49
calcium ion binding	14
iron ion binding	7
copper ion binding	6
cadmium ion binding	6
manganese ion binding	3

BEAS-2B. Significant transcripts were selected when expression values changed by 2-fold.

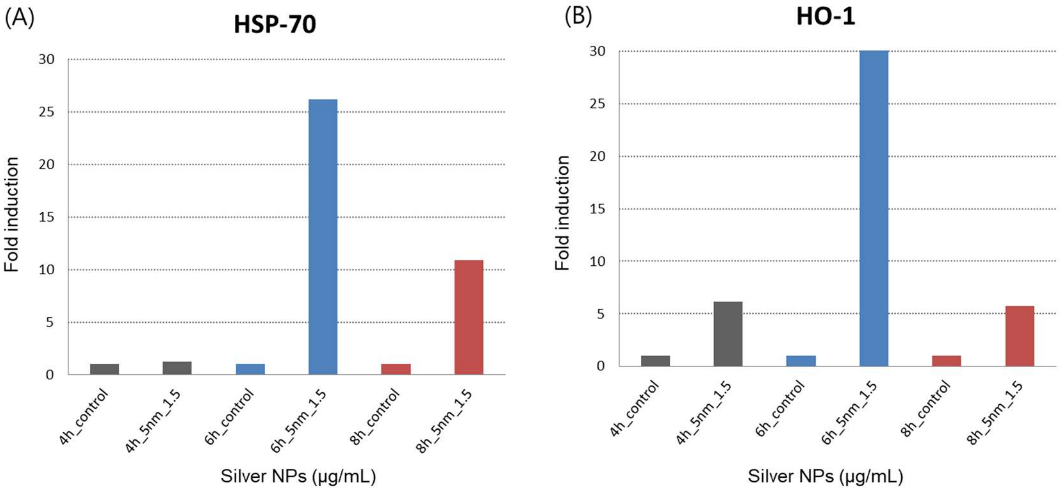

Figure S1. Changes in HSP-70 and HO-1 gene expression according to time course. (A), (B) RT-PCR analysis was performed for EA.hy926 cells treated with 5 nm silver NPs at 1.5 μg/mL for 4, 6, 8h. Control; RNase free water.

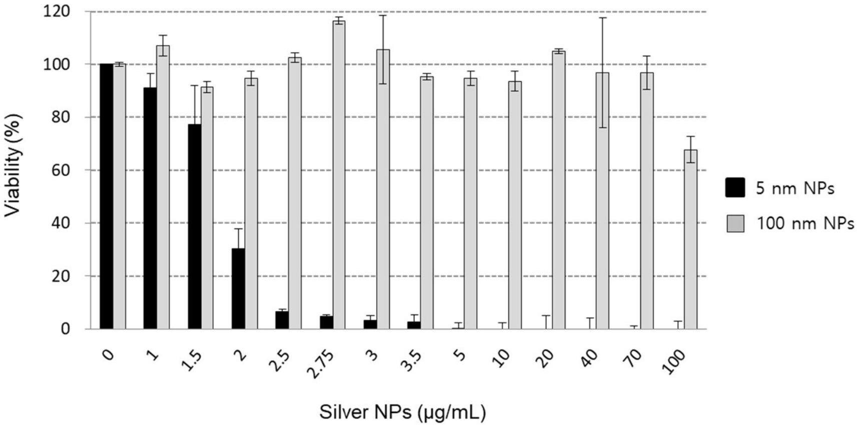

Figure S2. Viability of silver NPs in EA.hy926 cells. We have evaluated toxicity of 100 nm silver nanoparticles up to 100 μg/mL in which the cell viability was 68%. And LD_{50} of 100 nm was estimated as 118.3 μg/mL and 5 nm was 1.8 μg/mL.
